# Sedimentary Records of Organic Matter and Organochlorine Pesticides in a Eutrophic Shallow Plateau Lake: Implications for Eutrophication-Driven Pollutant Remobilization

**DOI:** 10.3390/toxics14080694

**Published:** 2026-08-06

**Authors:** Zhonghong Zhao, Li Bao, Min Ye, Naiming Zhang

**Affiliations:** 1 College of Plant Protection, Yunnan Agricultural University, Kunming 650201, China; zhaozhonghong168@126.com (Z.Z.); bbllty@163.com (L.B.); 2 College of Resources and Environment, Yunnan Agricultural University, Kunming 650201, China; 3 Yunnan Soil Fertility and Pollution Restoration Laboratory, Yunnan Agricultural University, Kunming 650201, China; 4 The Research Center for Smart Greenhouse Agriculture Engineering of Yunnan Provincial Universities, Yunnan Agricultural University, Kunming 650201, China

**Keywords:** organochlorine pesticides, eutrophication, sedimentary record, organic matter, remobilization, Lake Qilu

## Abstract

Lake eutrophication may promote the remobilization of historically buried organochlorine pesticides (OCPs), yet the underlying mechanisms remain unclear. This study reconstructed an 80-year sedimentary record from Lake Qilu to elucidate the linkages between organic matter evolution and OCP dynamics. Sedimentary proxies reveal a transition from oligo-mesotrophic conditions before the 1980s to eutrophic states since the 1990s, with significant increases in total organic carbon (TOC), dissolved organic matter (DOM), and hydrophilic organic matter (HIM), alongside δ^15^N enrichment from +2.0‰ to +11.3‰ and a positive δ^13^C shift from −21.7‰ to −14.5‰. The OCP record exhibits three distinct phases: accumulation during the 1940s–1980s, decline following regulatory bans during the 1990s–2000s, and anomalous resurgence during the 2010s–2020s, with ∑Drin (sum of aldrin, dieldrin, and endrin) reaching a historical peak of 10.54 μg/kg in the 2020s. Significant positive correlations were observed between both individual OCP congeners and total OCP classes and total nitrogen (TN), DOM and HIM (*p* < 0.01 or *p* < 0.05), indicating that eutrophication-driven algal proliferation releases hydrophilic organic matter, which forms soluble complexes with hydrophobic OCPs, facilitating their upward migration. This study provides direct field evidence for eutrophication-driven OCP remobilization, with important implications for risk assessment of pollutants in eutrophic lakes worldwide.

## 1. Introduction

Organochlorine pesticides (OCPs) are a class of persistent organic pollutants, mainly including DDT, HCH, chlordane, aldrin, dieldrin, and endosulfan. Due to their broad-spectrum insecticidal efficacy and low cost, they were widely used in global agriculture from the 1940s to the 1980s [1,2,3]. China banned the production and agricultural use of DDT and other organochlorine pesticides in 1983 and further tightened controls after ratifying the Stockholm Convention in 2001 [1,4,5]. Nevertheless, these compounds are still frequently detected in various environmental media such as soils, water bodies, and sediments, often at concentrations exceeding safety thresholds [6,7,8]. The primary sources of these pollutants include historical agricultural application and industrial emissions, long-range atmospheric transport and deposition, as well as surface runoff inputs from urban and intensively cultivated agricultural areas [1,3,5,6]. The long-term environmental persistence of OCPs is primarily attributed to their high hydrophobicity, strong resistance to degradation, and high affinity for particulate organic matter, which enable their prolonged sequestration in sediments [9,10,11,12]. However, under certain environmental conditions, historically buried OCPs may be remobilized, posing sustained ecological risks to benthic organisms and the overlying water column [13,14,15,16]. To date, the post-depositional behavior of these legacy pollutants, particularly whether eutrophication acts as a driving force for their remobilization, remains poorly understood.

Eutrophication, resulting from excessive nutrient inputs via agricultural runoff, domestic wastewater, and atmospheric deposition, has emerged as a widespread environmental problem in lakes globally [17,18,19,20]. The settlement and decomposition of algal blooms not only alter the quantity and composition of sedimentary organic matter (SOM) but also cause notable biogeochemical changes at the sediment–water interface (SWI), such as oxygen depletion and redox potential shifts [17,21,22,23,24]. These eutrophication-induced sedimentary changes can significantly affect the fate of hydrophobic organic contaminants (HOCs) in aquatic ecosystems [9,25,26,27]. Shifts between macrophyte-dominated and algae-dominated states alter the quality and quantity of settling organic matter, thereby affecting the sorption of HOCs onto sediments [8,9,14,28]. In shallow lakes, frequent sediment resuspension promotes the exchange of HOCs between sediment and water, whereas in deep stratified lakes, permanent burial of HOCs is favored [7,8,12]. Elevated sediment total organic carbon (TOC) contents enhance the sorption of hydrophobic pesticides, and increased phytoplankton-derived OM inputs during eutrophication promote the burial of HOCs in lake sediments [9,28]. Conversely, hydrodynamic disturbances can remobilize sediment-associated pesticides into the water phase, and sediment resuspension has been recognized as a major secondary source of OCPs [29]. However, direct field evidence connecting the historical course of eutrophication to the vertical redistribution of OCPs in lake sediments remains lacking.

Lake Qilu, situated in Yunnan Province, is a representative shallow eutrophic lake in southwestern China. Over the past several decades, its trophic state has deteriorated from oligotrophic to highly eutrophic as a consequence of intensive agricultural inputs, rapid urbanization, and growing tourism pressure. Although existing research has focused predominantly on nutrient dynamics and phytoplankton bloom succession, the long-term depositional history of OCPs and its response to eutrophication-driven alterations in organic matter remain largely unaddressed. To fill this knowledge gap, this study utilizes high-resolution sediment core records with three primary objectives: (1) to track the historical trajectories of organic matter composition and source apportionment from the 1940s to the 2020s; (2) to determine the temporal variation and phase-specific characteristics of individual OCP compounds; and (3) to unravel the driving mechanisms by which organic matter properties mediate OCP mobilization and accumulation throughout the eutrophication gradient. This work is anticipated to contribute to the sustainable management of persistent organic pollutants in lake environments.

## 2. Materials and Methods

### 2.1. Study Area

Lake Qilu is located in Tonghai County, Yunnan Province, with a surface area of approximately 36.73 km^2^. It is a typical shallow eutrophic lake on the Yunnan–Guizhou Plateau [7]. The catchment has a subtropical monsoon climate, with a mean annual temperature of 15.6 °C and mean annual precipitation of approximately 945 mm. Land use within the watershed is predominantly characterized by intensive agricultural cultivation, particularly vegetable and flue-cured tobacco farming, which has been identified as the primary source of agricultural non-point source pollution [7]. Historical agricultural practices have contributed substantially to organochlorine pesticide (OCP) residues in the lake, with pesticides primarily transported via surface runoff and atmospheric dry/wet deposition [7]. In addition to agricultural pressures, the Qilu Lake watershed faces multiple environmental challenges, including progressive lake area shrinkage, aquatic ecosystem degradation, and persistent eutrophication.

### 2.2. Sample Collection

**Figure 1:**
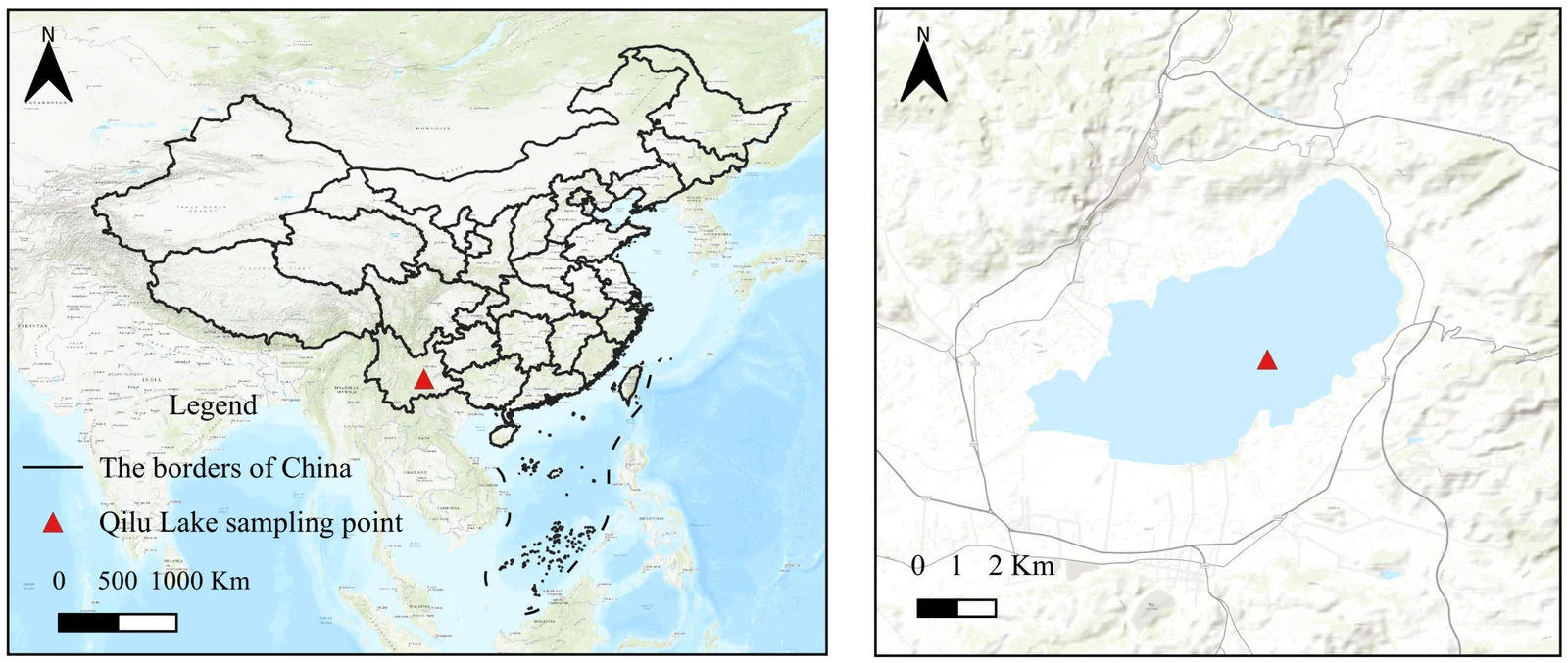
Sediment core sampling location in Qilu Lake.

In 2023, a single sediment core was collected from the deepest depositional area of Lake Qilu (water depth ~3.7 m) using a gravity corer (Figure 1). The core, spanning 0–30 cm in length, was sectioned at 2 cm intervals, yielding a total of 15 samples. The samples were placed in tin foil bags and immediately frozen at −20 °C. All samples were subsequently freeze-dried (−80 °C, Boyikang FD-1B-80, Beijing, China) for one week until completely dry. The dried sediments were ground with a ceramic mortar, passed through a 100-mesh nylon sieve, homogenized, stored in tin foil bags, and kept at room temperature in a desiccated environment for further analysis.

### 2.3. Sedimentary Organic Matter and Stable Carbon–Nitrogen Isotope Analysis

The chronological framework was established by correlating high-resolution geochemical stratigraphy with a reference core that had been reliably dated by ^210^Pb and ^137^Cs [30]. A time-lag correction was applied to account for the difference in sampling intervals between the reference core (collected in 2014) and the present study.

Total organic carbon (TOC) in sediments was determined using a TOC auto-analyzer [31]. Total nitrogen (TN) was measured using the alkaline potassium persulfate digestion–ultraviolet spectrophotometric method [32]. Stable carbon and nitrogen isotopes (δ^13^C and δ^15^N) were analyzed using an elemental analyzer–stable isotope ratio mass spectrometer (EA-IRMS, Bremen, Germany).

Sedimentary organic matter fractionation was performed following the procedures recommended by the International Humic Substances Society (IHSS) with modifications [33]. Organic carbon contents of each fraction were determined using a TOC analyzer.

### 2.4. Extraction and Instrumental Analysis of Organochlorine Pesticides

Freeze-dried sediment samples were homogenized with activated quartz sand and extracted using accelerated solvent extraction (ASE 350, Dionex, Sunnyvale, CA, USA) with dichloromethane. The extracts were concentrated by rotary evaporation, purified on a silica gel–alumina column (2:1, *w*/*w*), and eluted with n-hexane/dichloromethane (7:3, *v*/*v*). After solvent exchange and further concentration, the final extract was adjusted to 1 mL and stored at −20 °C until analysis.

Quantification was performed using a gas chromatograph (Agilent 7890A, Santa Clara, CA, USA) equipped with a ^63^Ni micro-electron capture detector (μECD). Separation was achieved on an HP-5MS capillary column (30 m × 0.25 mm × 0.25 μm) with helium as the carrier gas at 1.5 mL/min and high-purity nitrogen (60 mL/min) as the makeup gas. The injector and detector temperatures were 250 °C and 320 °C, respectively, with a 1 μL splitless injection. Peak identification was confirmed by GC-MS in full scan (*m*/*z* 45–500) and selected ion monitoring modes.

### 2.5. Quality Assurance and Quality Control (QA/QC)

OCP peaks were identified by comparison with authentic standards and quantified using an internal standard method (TCMX). Calibration curves (R^2^ = 0.992–0.999) and six replicate injections (RSDs = 0.58–6.93%) demonstrated good reproducibility, with retention times recalibrated every 10 samples.

Method detection limits (MDLs) ranged from 0.003 to 0.086 ng/g (S/N = 3). No target analytes were detected in laboratory blanks. Spike recoveries in diatomaceous earth ranged from 79% to 110%, meeting USEPA requirements.

The 20 target OCPs included α-HCH, β-HCH, γ-HCH, δ-HCH, heptachlor, aldrin, heptachlor epoxide, γ-chlordane, α-chlordane, α-endosulfan, p,p′-DDE, dieldrin, endrin, β-endosulfan, p,p′-DDD, endrin aldehyde, endosulfan sulfate, p,p′-DDT, endrin ketone, and methoxychlor. MDLs were 0.003–0.086 ng/g, blanks were free of contaminants, and spike recoveries (79–110%) met USEPA requirements, except for slightly lower recoveries of p,p′-DDT (69%) and methoxychlor (71%).

## 3. Results and Discussion

### 3.1. Composition and Source Evolution of Sedimentary Organic Matter

Lake sedimentary organic matter serves as an important proxy for reflecting changes in lake environments and the intensity of anthropogenic activities within catchments [34,35,36]. In this study, fulvic acid (FA) was further fractionated into hydrophilic fulvic acid (HIFA) and hydrophobic fulvic acid (HOFA), and dissolved organic matter (DOM) was partitioned into hydrophilic organic matter (HIM) and hydrophobic organic matter (HOM). By analyzing the content variations in total organic carbon (TOC), FA and its hydrophilic/hydrophobic fractions (HIFA and HOFA), as well as DOM and its hydrophilic/hydrophobic fractions (HIM and HOM), combined with the tracing information of δ^13^C and δ^15^N, the source structure and historical evolution of lake organic matter can be effectively reconstructed [35,36]. Specifically, HIFA and HIM are primarily derived from autochthonous sources (e.g., algae and aquatic plants) with high bioavailability, whereas HOFA and HOM are mainly derived from allochthonous terrestrial higher plant inputs and are relatively resistant to microbial degradation [33,36]. The sedimentary record of organic matter in Lake Qilu over the past 80 years clearly reveals a transition from a natural state to an eutrophic state.

**Figure 2:**
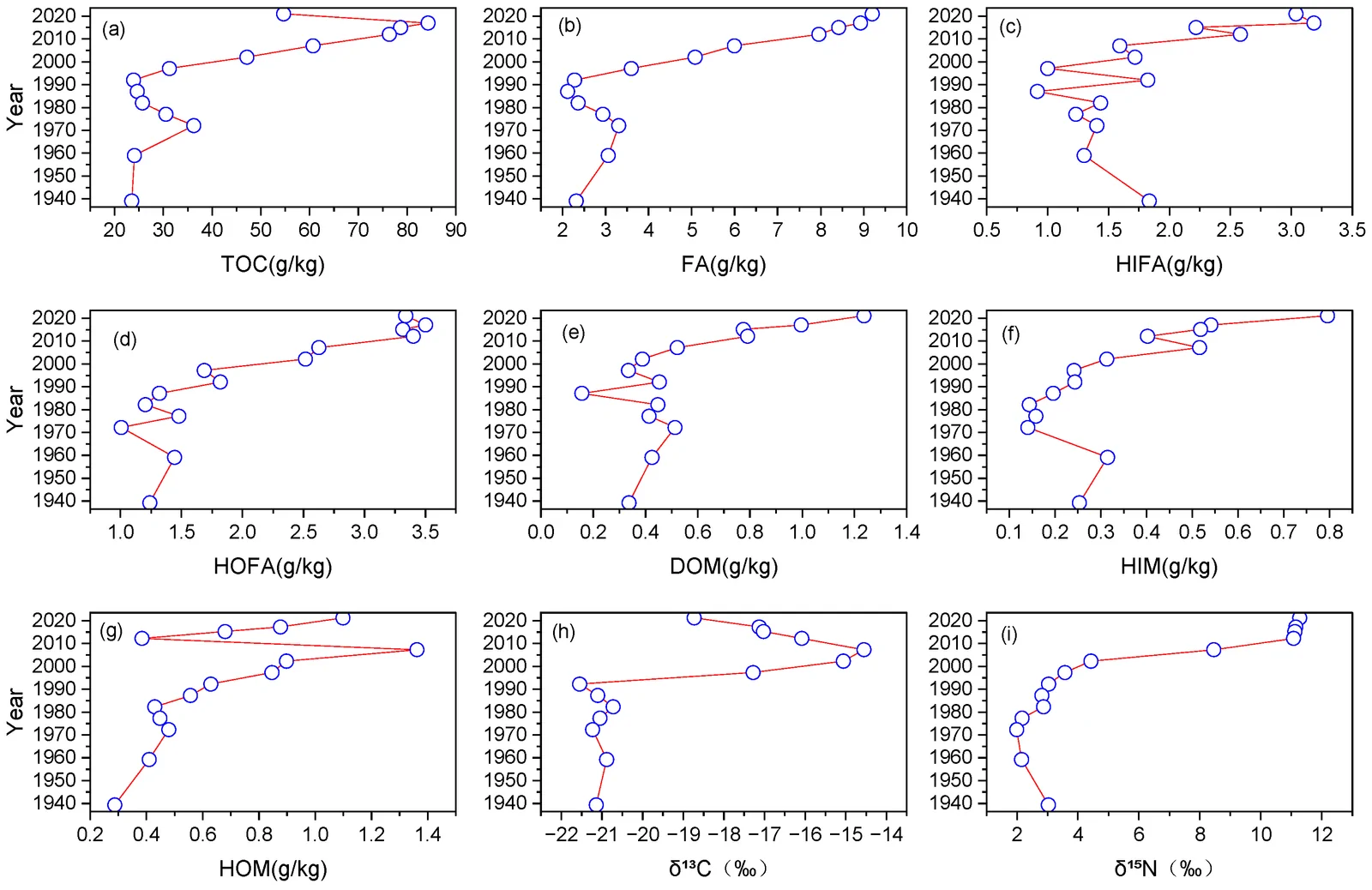
Historical records of organic matter composition and stable carbon and nitrogen isotopes (δ^13^C and δ^15^N) in the sediment core from Lake Qilu: (**a**) total organic carbon (TOC); (**b**) fulvic acid (FA); (**c**) hydrophilic fulvic acid (HIFA); (**d**) hydrophobic fulvic acid (HOFA); (**e**) dissolved organic matter (DOM); (**f**) hydrophilic organic matter (HIM); (**g**) hydrophobic organic matter (HOM); (**h**) stable carbon isotope (δ^13^C); (**i**) stable nitrogen isotope (δ^15^N).

During 1939–1987, TOC contents in Lake Qilu sediments were generally low, fluctuating between 23.54 and 36.27 g/kg, with a mean value of approximately 27.5 g/kg. FA contents remained at low levels of 2.13–3.31 g/kg, and DOM was only 0.16–0.51 g/kg (Figure 2a,b,e), reflecting low lake primary productivity and limited external organic matter inputs. Similarly, HIFA and HOFA, as well as HIM and HOM, all remained at relatively low and stable levels during this period (Figure 2c,d,f,g). δ^13^C values were stable between −21.7‰ and −20.7‰ (Figure 2h), closely approaching the mixed end-member between typical terrestrial C3 plant δ^13^C signatures and lacustrine autochthonous organic matter signatures [37,38,39,40]. Meanwhile, δ^15^N remained within a narrow range of +2.0‰ to +3.0‰ (Figure 2i), falling within the typical range of natural soils and lakes without significant anthropogenic disturbance [41,42,43]. During this stage, the ratio of hydrophobic (HOM) to hydrophilic (HIM) DOM remained relatively stable, indicating that the source composition of organic matter did not undergo significant changes and that microbial degradation and transformation were weak [36,44,45]. Collectively, these lines of evidence indicate that the primary source of organic matter in Lake Qilu during this stage was terrestrial vegetation debris carried by surface soil erosion from the catchment, whereas the lake’s own primary productivity contributed very little.

During 1992–2007, TOC contents in Lake Qilu sediments increased dramatically to 60.74 g/kg. During this period, FA increased from 2.28 g/kg to 6.00 g/kg, and DOM increased from 0.45 g/kg to 0.52 g/kg (Figure 2a,b,e). Among the FA sub-fractions, HIFA initially decreased and then rebounded, whereas HOFA showed a sustained increase over the entire period. For the DOM sub-fractions, both HIM and HOM increased substantially, with HIM nearly doubling (Figure 2c,d,f,g). δ^13^C increased from −21.544‰ to −14.547‰, while δ^15^N continuously rose from +3.06‰ to +8.48‰. During the eutrophication process, massive algal proliferation leads to an increase in the δ^13^C of dissolved inorganic carbon in the water column, which consequently results in a positive shift in the δ^13^C of sedimentary organic matter [37,38,39,40,46,47]. The δ^13^C reached a peak of −14.54‰ in 2007, a value very close to the end-member of pure phytoplankton origin, indicating that autochthonous algal contributions far exceeded terrestrial inputs [37,38,48]. Meanwhile, the continuous increase in δ^15^N directly reflected the significant enhancement of exogenous anthropogenic nitrogen inputs [39,40,41]. The enrichment of δ^15^N is typically associated with fertilizer application, domestic sewage discharge, and livestock wastewater inputs, which themselves possess high δ^15^N signatures [42,43]. In the Lake Qilu catchment, intensive agricultural cultivation (particularly vegetable and flue-cured tobacco farming) and untreated domestic sewage from surrounding towns are the predominant sources of anthropogenic nitrogen, consistent with the land use characteristics of this region [7]. In addition to these external inputs, other factors such as hydrological exchange, internal nutrient loading from sediment release, and enhanced denitrification under eutrophic conditions may also contribute to the progressive δ^15^N enrichment observed in the sedimentary record [18,30]. After entering the lake, these anthropogenic nitrogen sources undergo further fractionation through algal assimilation and denitrification, progressively elevating the δ^15^N in sediments [44]. During this stage, the significant increase in the HIM/HOM ratio further demonstrated the dominant role of increasing algal-derived organic matter [36,45,46].

After entering the 2010s, all indicators of sedimentary organic matter in Lake Qilu have stabilized at high levels, indicating that the lake has fully entered a eutrophic state [34,35,36,46]. Although TOC decreased to 54.69 g/kg in 2021 after reaching a historical peak of 84.32 g/kg in 2017, it remained far above historical baseline levels [34,35]. In contrast, FA and DOM continuously increased to their historical maxima (9.20 g/kg and 1.24 g/kg) [36,44]. Among the FA sub-fractions, HIFA increased substantially and remained at elevated levels, while HOFA stabilized after a moderate increase. For the DOM sub-fractions, HIM showed a gradual upward trend, whereas HOM decreased notably after reaching its peak in 2007 and then remained relatively stable (Figure 2c,d,f,g). These divergent trends suggest that although autochthonous algal contributions continued to grow, the hydrophobic components (HOFA and HOM) exhibited different response patterns during this eutrophic stage. δ^15^N further increased and stabilized between +11.0‰ and +11.3‰, exceeding the normal range of δ^15^N for natural lakes [41,43]. This feature clearly indicates the continuous input of high-intensity anthropogenic nitrogen sources, promoting progressive enrichment of ^15^N in the nitrogen pool, which is ultimately recorded in the sediments [39,40,42]. δ^13^C showed a negative shift from its peak value of −14.54‰ in 2007 to approximately −18.72‰ in 2021, which may be related to the massive decomposition of sedimentary algae and the enhanced metabolic activity of heterotrophic microorganisms [44,45,47]. Nevertheless, the δ^13^C value of −18.7‰ remains significantly higher than the −21‰ level during the early terrestrial-dominated period, indicating that autochthonous algae remain the absolute dominant component of sedimentary organic matter [37,38,48].

### 3.2. Historical Deposition Characteristics of Organochlorine Pesticides in Sediment Core

**Figure 3:**
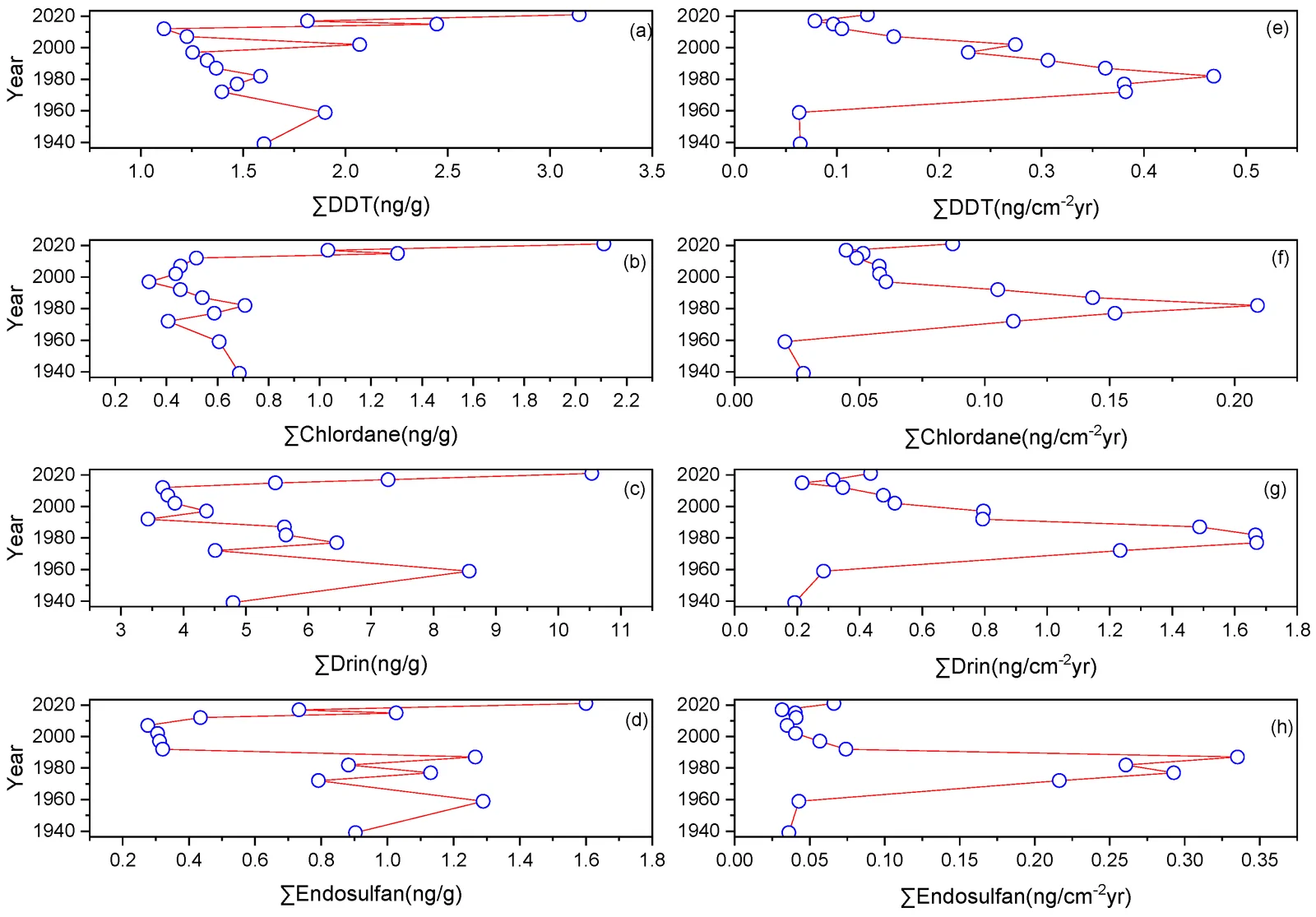
Historical sedimentary records of OCP compound classes in the sediment core. Panels (**a**–**d**) show the concentration profiles of ∑DDT, ∑Chlordane, ∑Drin (sum of aldrin, dieldrin, and endrin), and ∑Endosulfan, respectively, while panels (**e**–**h**) show the corresponding sedimentary flux profiles of the four OCP classes. The deposition flux trends illustrated in panels (**e**–**h**) reflect the historical variations in OCP accumulation rates in the core sediments of Lake Qilu over the past eight decades.

Although the production and agricultural application of OCPs have been banned in China since 1983, their residues remain detectable in various environmental media due to their persistence, posing ongoing ecological risks. The concentrations of OCPs in the sediment cores of Lake Qilu have recorded the history of pesticide use in this catchment over the past eight decades (Figure 3a–d). In the 1940s, the sedimentary layer contained ΣDDT at 1.60 ng/g, ΣChlordane at 0.69 ng/g, ΣDrin (sum of aldrin, dieldrin, and endrin) at 4.80 ng/g, and ΣEndosulfan at 0.90 ng/g. During this period, OCPs were just beginning to be applied in agriculture on a large scale globally. The insecticidal properties of DDT were discovered in the late 1930s, and it was rapidly promoted as a revolutionary insecticide after World War II; chlordane and aldrin also entered the market successively in the mid-1940s [25,49]. Therefore, OCPs in the 1940s sediments primarily reflected background inputs from early application and long-range atmospheric transport [8,26,50]. By the 1950s–1960s, the concentrations of all components showed significant increases: ΣDDT rose to 1.90 ng/g, ΣChlordane slightly decreased to 0.61 ng/g, ΣDrin increased sharply to 8.57 ng/g (1.8 times the 1940s level), and ΣEndosulfan rose to 1.29 ng/g (Table 1). This significant increase is fully consistent with the historical context of China’s large-scale promotion of OCPs since the 1950s to ensure food security [2,25,50,51,52]. Notably, ΣDrin (aldrin and dieldrin) dominated the OCP composition in Lake Qilu (8.57 ng/g), indicating that the catchment may have focused on the control of soil-dwelling pests during the early stage of agricultural development or exhibited a specific preference for the application of aldrin-type pesticides [26,50]. The high values of ΣDrin in the 1950s also reflected the widespread global use of aldrin and dieldrin as broad-spectrum soil insecticides after World War II [49,53].

During the 1970s, the concentrations of each component remained relatively stable: ∑DDT ranged from 1.40 to 1.47 ng/g, ∑Chlordane from 0.41 to 0.59 ng/g, ∑Drin from 4.51 to 6.45 ng/g, and ∑Endosulfan from 0.79 to 1.13 ng/g, with ∑Drin still being the major contributor. In the 1980s, the sedimentary record showed significant changes—in the early 1980s, the concentrations of each component were ∑DDT 1.59 ng/g, ∑Chlordane 0.71 ng/g, ∑Drin 5.64 ng/g, and ∑Endosulfan 0.88 ng/g; by the late 1980s, ∑Drin slightly decreased to 5.62 ng/g, ∑DDT decreased to 1.37 ng/g, ∑Chlordane decreased to 0.54 ng/g, while ∑Endosulfan increased significantly to 1.27 ng/g. Compared with the policy timeline of China’s official ban on DDT and other OCPs in 1983, the post-ban peak of ∑Endosulfan can be attributed to the fact that endosulfan, as a broad-spectrum insecticide, was not banned simultaneously with DDT in China and its use continued until the late 2000s [54,55,56]. Therefore, the endosulfan peak in the 1980s likely reflected the historical fact that endosulfan was widely promoted as a DDT substitute during this period [51,54]. Meanwhile, ∑Drin remained at high levels of 5.62–5.64 ng/g after the ban, with no significant attenuation compared with the 1970s levels, further confirming the extremely high persistence of aldrin and dieldrin in sedimentary environments [26,52,57].

Entering the 1990s, the concentrations of all components decreased significantly: ∑DDT ranged from 1.25 to 1.33 ng/g, ∑Chlordane from 0.33 to 0.46 ng/g, ∑Drin from 3.43 to 4.37 ng/g, and ∑Endosulfan from 0.31 to 0.32 ng/g. The 2000s continued this low-level trend: ∑DDT ranged from 1.23 to 2.07 ng/g, ∑Chlordane from 0.44 to 0.46 ng/g, ∑Drin from 3.75 to 3.86 ng/g, and ∑Endosulfan from 0.28 to 0.31 ng/g. This significant decline was directly attributable to China’s progressively strengthened control over OCPs since the 1980s—DDT and its metabolites were banned in 1983, followed by chlordane, aldrin, and dieldrin being successively listed as restricted or prohibited substances [1,4,25,50]. After China signed the Stockholm Convention in 2001, control over persistent organic pollutants was further intensified [4,8,49]. Notably, although all components were at historical low levels, ∑Drin (3.43–4.37 ng/g) remained significantly higher than ∑DDT (1.23–2.07 ng/g), continuing the pollution characteristic of aldrin-type dominance in this catchment [26,50,57]. ∑Endosulfan decreased to 0.28–0.32 ng/g during the 1990s–2000s, reaching its historical minimum, indicating that endosulfan use was effectively controlled during this period. However, ∑Drin remained above 3.5 ng/g nearly two decades after the ban, indicating that the degradation rates of aldrin and dieldrin are extremely slow, with half-lives of up to decades in anoxic sedimentary environments [26,52].

The period from the 2010s to the 2020s represents the most anomalous and highly concerning stage in the history of OCP pollution in Lake Qilu. In the early 2010s, the concentrations of each component were ∑DDT 1.11 ng/g, ∑Chlordane 0.52 ng/g, ∑Drin 3.67 ng/g, and ∑Endosulfan 0.43 ng/g; in the mid-2010s, a comprehensive rebound occurred to ∑DDT 2.45 ng/g, ∑Chlordane 1.30 ng/g, ∑Drin 5.47 ng/g, and ∑Endosulfan 1.03 ng/g; by the 2020s, concentrations surged to historical peaks: ∑DDT 3.14 ng/g, ∑Chlordane 2.11 ng/g, ∑Drin 10.54 ng/g, and ∑Endosulfan 1.60 ng/g, with all components reaching or approaching their highest recorded levels [7,30,58].

The sedimentary flux trends of OCPs in Lake Qilu sediments indicate that the fluxes of all components increased significantly from the 1940s to the 1980s (Figure 3e–h), reaching historical peaks during the 1970s–1980s (∑Drin up to 1.67 ng·cm^−2^·yr^−1^, ∑DDT up to 0.47 ng·cm^−2^·yr^−1^), followed by a significant decline during the 1990s–2000s, but rebounding again during the 2010s–2020s. This trend is closely linked to China’s pesticide use history and regulatory policies: the high fluxes during the 1970s–1980s reflected the large-scale agricultural application of OCPs, while the significant decline after the 1983 ban on DDT and other OCPs demonstrated the effectiveness of policy interventions [50,51,59,60,61,62]. However, the rebound of ∑Drin flux to 0.44 ng·cm^−2^·yr^−1^ and ∑Chlordane flux to 0.09 ng·cm^−2^·yr^−1^ in the 2020s may be related to recent extreme precipitation events intensifying soil erosion and releasing historically accumulated OCP reserves, as well as land use changes (such as urbanization and reforestation) disturbing surface stability [13,30,52,61]. The rebound of some components may also be influenced by the “concentration effect” caused by decreased sedimentation rates, but the increase in absolute fluxes still indicates recent new inputs or secondary releases [8,26]. Similar post-ban resurgence patterns have been observed in other lake systems. For example, OCP fluxes in sediments from the Tibetan Plateau rebounded during flood events due to remobilization of historical residues [13]. This anomalous rebound suggests that although prohibition policies have effectively controlled new uses of OCPs, the secondary release of historical residues is becoming a persistent threat to lake contamination [13,30,52,61].

**Figure 4:**
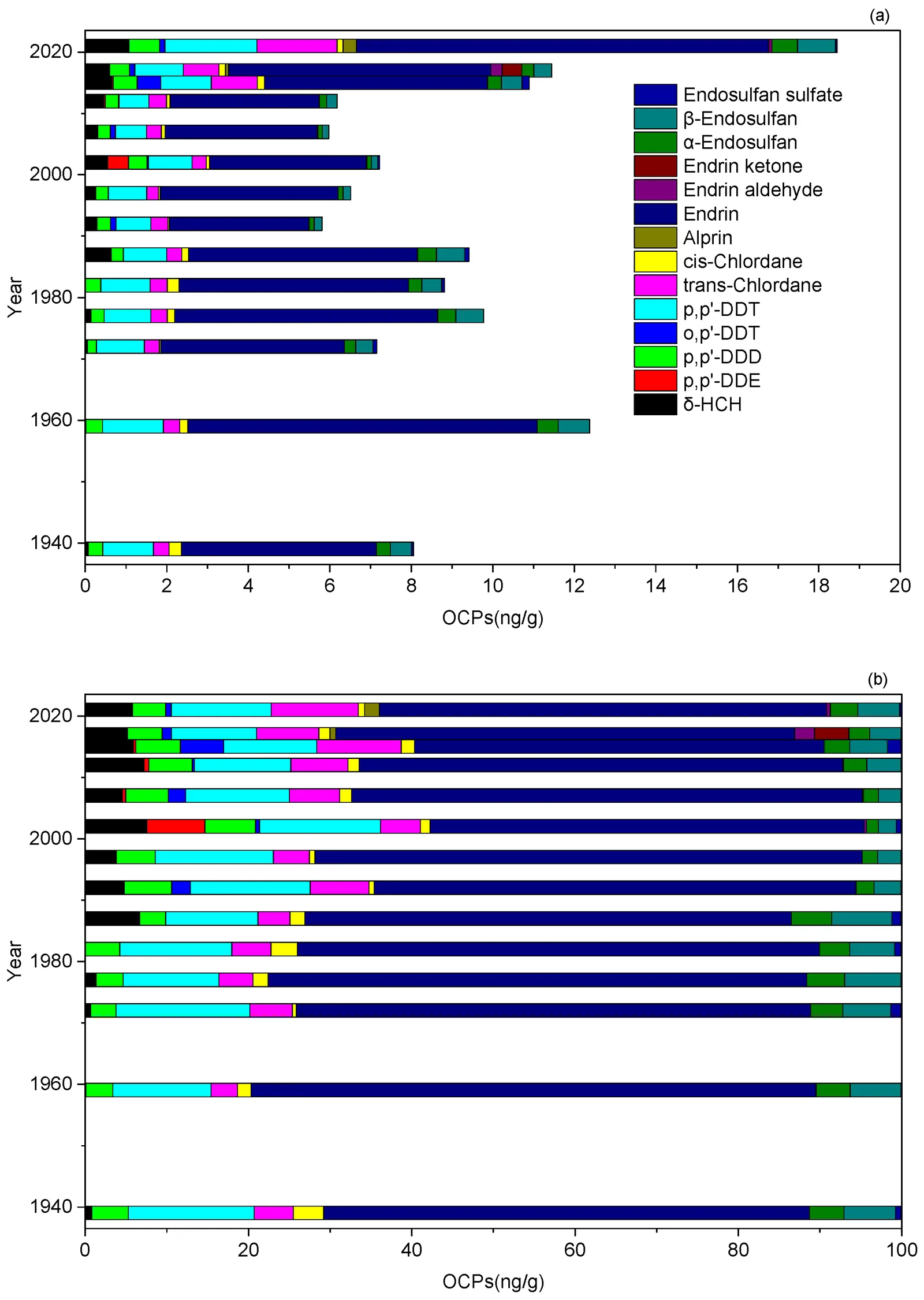
Temporal profiles of individual OCP compound concentrations in the sedimentary record of Lake Qilu. Panels (**a**) and (**b**) present the absolute concentrations and relative abundances of individual OCP compounds over the core depth, respectively.

The historical compositional patterns of individual OCP congeners in Lake Qilu sediments provide further insights into their sources and degradation status (Figure 4). For the ∑Drin group, endrin consistently dominated the composition throughout the sediment core, accounting for 50.2–69.3% of the total (Figure 4b), whereas aldrin was rarely detected (mostly below detection limits). This indicates that endrin, rather than aldrin, was the primary compound applied in this catchment. δ-HCH was detected in 93.3% of the sediment core samples (13 out of 14 samples), with proportions ranging from 0.08% to 7.54% of total OCPs, whereas the other HCH isomers (e.g., β-HCH and γ-HCH) were consistently below the method detection limits in all samples.

**Figure 5:**
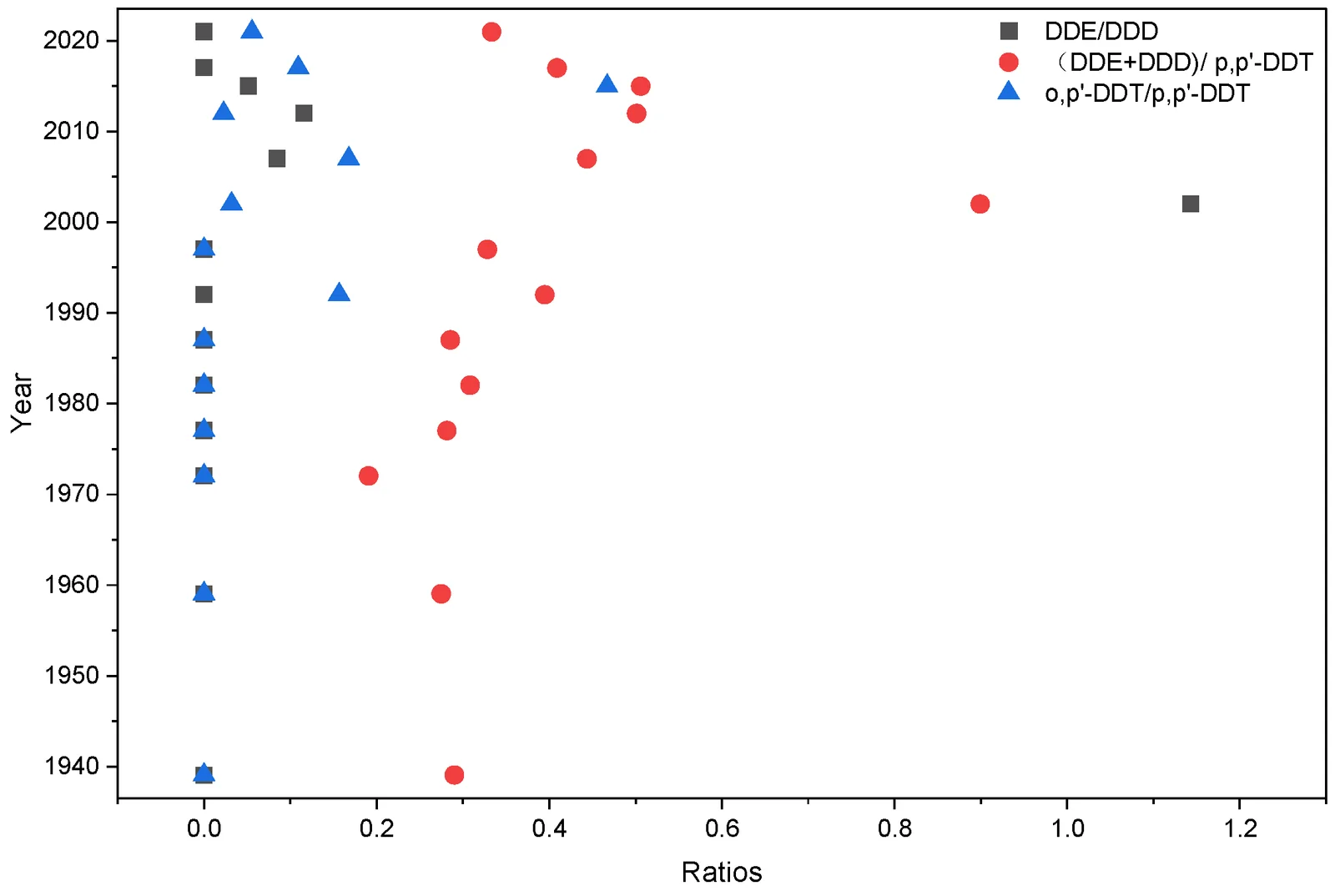
Scatter plot distribution of diagnostic ratios of DDT and its metabolites (DDTs) in the core sediments of Lake Qilu.

For the ∑DDT group, p,p′-DDT was the predominant congener, comprising 10.4–16.4% of total OCPs (Figure 4a). The (DDE + DDD)/p,p′-DDT ratios varied from 0.19 to 0.90 (Figure 5), with low values before the 1980s (0.19–0.39) indicating relatively fresh DDT inputs during the period of active agricultural use and elevated ratios in the 2000s (up to 0.90 in 2002) suggesting ongoing degradation of historical DDT residues after the ban [56,63]. The o,p′-DDT/p,p′-DDT ratios remained below 0.5 throughout the record, indicating that the DDT sources were primarily from technical DDT rather than dicofol [26,64,65]. The DDE/DDD ratios were consistently zero throughout most of the core, with the sole exception of a value of 1.14 in 2002, indicating that the degradation of p,p′-DDT occurred predominantly via the anaerobic pathway (yielding DDD). This is consistent with the reducing conditions associated with long-term eutrophication and bottom-water hypoxia [63,64,65]. The anomalous DDE/DDD peak in 2002 may reflect a transient oxygenation event at the sediment–water interface or the input of weathered DDT residues from catchment soils.

### 3.3. Mechanisms of Eutrophication-Driven Mobilization and Transport of Organochlorine Pesticides in Sediment Core

Sedimentary records from Lake Qilu reveal a critical environmental coupling process by which eutrophication drives the remobilization of historically buried OCPs. Since the intensification of lake eutrophication in the 1990s, sediment organic matter, particularly DOM, has increased markedly (Figure 2). Concurrently, after a decline from the 1990s to the 2000s, the concentrations of multiple OCP congeners exhibited a widespread resurgence in the 2010s. This spatiotemporal synchronicity implies an intrinsic linkage between eutrophication and OCP remobilization.

**Figure 6:**
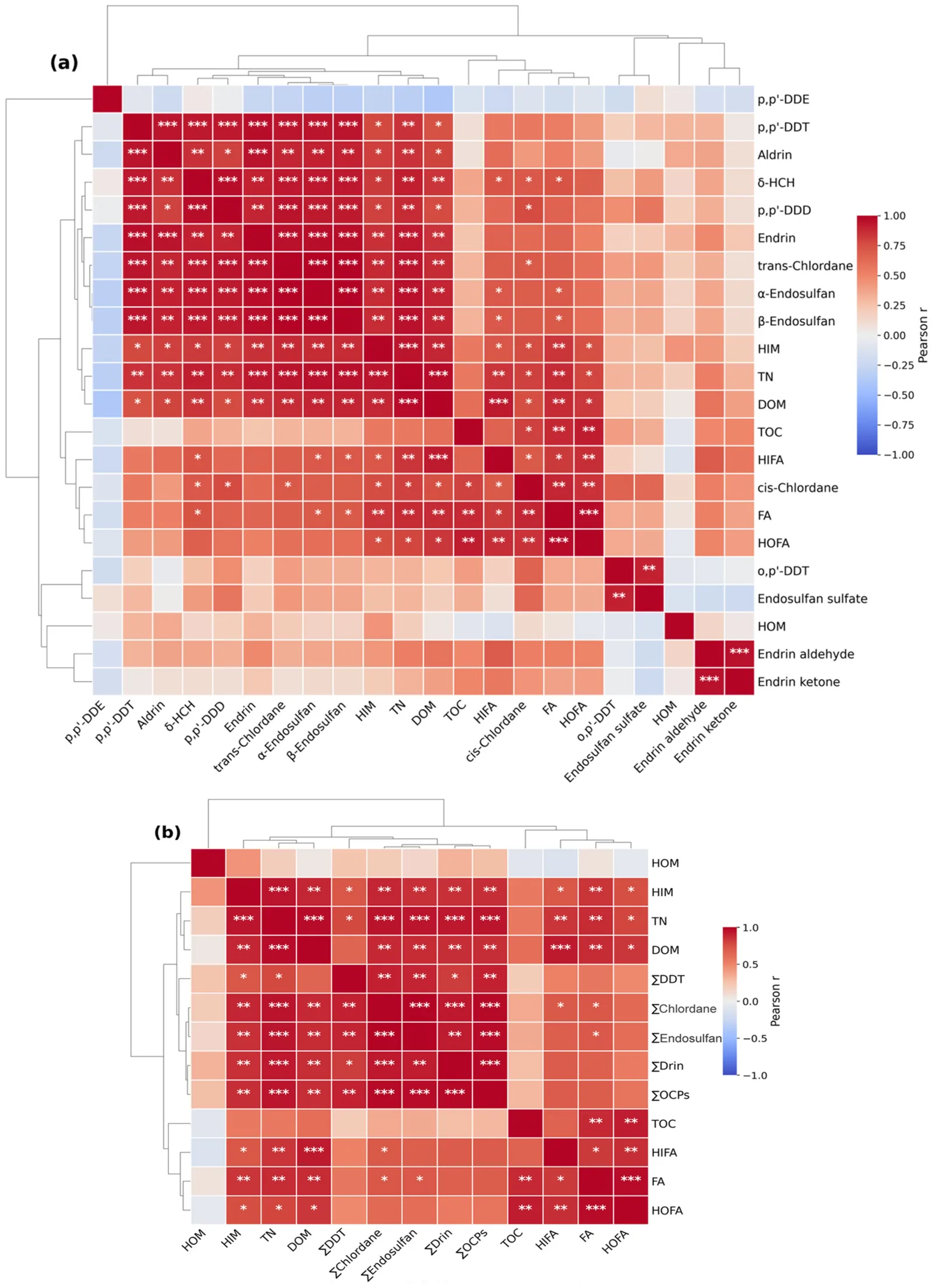
Correlation analysis between organochlorine pesticides (OCPs) and organic matter fractions in the sediment core: (**a**) individual OCP compounds; (**b**) total concentrations of each OCP class. Significance levels are indicated as follows: * *p* < 0.05, ** *p* < 0.01, *** *p* < 0.001.

Further Pearson correlation analyses between sediment OCP components and OM fractions provide robust statistical support for this linkage. For individual OCP compounds (Figure 6a), δ-HCH, aldrin, endrin, trans-chlordane, cis-chlordane, α-endosulfan, β-endosulfan, and p,p′-DDD all exhibited significant positive correlations with TN, DOM, and HIM (*p* < 0.05 or *p* < 0.01). Notably, HIM demonstrated exceptionally strong correlations with most OCP compounds, particularly with trans-chlordane, endrin, and endosulfan isomers. In contrast, hydrophobic organic matter (HOM and HOFA) showed no significant correlations with any OCP compounds (all *p* > 0.05), indicating that the hydrophilic fractions of organic matter, rather than the hydrophobic fractions, play a dominant role in OCP mobilization.

For the total concentrations of each OCP class (Figure 6b), similar patterns were observed. ∑OCP, ∑DDT, ∑Chlordane, ∑Drin, and ∑Endosulfan all showed significant positive correlations with TN, HIM, and DOM (*p* < 0.05 or *p* < 0.01). Among these, ∑OCP, ∑Chlordane, ∑Drin and ∑Endosulfan exhibited the strongest correlations (*p* < 0.01 or *p* < 0.001), while ∑DDT showed relatively weaker but still significant correlations (*p* < 0.05). The consistently strong correlations between OCPs and TN further suggest that enhanced nitrogen loading, a key driver of eutrophication, may indirectly promote OCP remobilization through the production of hydrophilic organic carriers.

Studies have shown that algal bloom-derived DOM, rich in aromatic and carboxyl functional groups, can preferentially adsorb hydrophobic pollutants such as polycyclic aromatic hydrocarbons, thereby influencing their burial behavior in sediments [9,28,59,60,61]. Since the acceleration of eutrophication in Lake Qilu, massive algal proliferation has produced substantial OM, which upon degradation releases abundant hydrophilic compounds rich in polar functional groups such as carboxyl and hydroxyl moieties [66,67,68]. On one hand, these HIM fractions, through their polar functional groups, can form water-soluble complexes or colloidal associations with hydrophobic OCPs, significantly enhancing the apparent solubility of OCPs in pore water [9,69]. On the other hand, these small molecular organic moieties are more prone to diffusive and convective transport across the sediment–water interface (SWI), carrying the complexed OCPs upward from deep pore water to the overlying water, ultimately leading to their redistribution and enrichment in surface sediments [10,13,60].

The eutrophication-driven remobilization of OCPs brings previously sequestered pollutants back into direct contact with benthic organisms and the overlying water column, substantially elevating ecological exposure risks [13,14,15,16,28]. Hypoxia caused by eutrophication alters redox conditions at the SWI, potentially facilitating OCP release from sediment to water [70]. Likewise, increased HIM may promote OCP mobilization through complexation mechanisms [71,72,73]. OCPs associated with HIM are particularly susceptible to diffusive transport into the overlying water via pore water exchange, subsequently entering the aquatic food web and exerting potential ecotoxicological effects [74,75]. Of particular concern is the significant positive correlation between ∑Drins—compounds with high ecological risk—and both DOM and HIM in Lake Qilu, which further underscores that eutrophication may enhance the exchange flux of these pesticides across the SWI.

## 4. Conclusions

This study reconstructed an 80-year sedimentary record of organic matter and OCPs in Lake Qilu and examined the links between eutrophication and OCP mobilization. Three main conclusions are drawn:

(1) The sedimentary organic matter profile shows a clear shift from natural to eutrophic conditions: 1939–1987, oligotrophic to mesotrophic with terrestrial organic matter; 1992–2007, rapid eutrophication due to anthropogenic nutrient loading; since 2010, all proxies remain elevated, indicating a hypereutrophic state dominated by algal-derived organic matter.

(2) The OCP record exhibits three distinct phases: progressive accumulation from the 1940s to the 1980s, marked decline during the 1990s–2000s following regulatory bans, and widespread resurgence from the 2010s to the 2020s, with all compounds peaking in the most recent decade. ∑Drin consistently dominated throughout, highlighting the strong persistence of aldrin and dieldrin under anoxic conditions.

(3) Pearson correlation analysis shows significant positive correlations between OCPs and organic matter parameters (*p* < 0.05 or *p* < 0.01), supporting a mobilization mechanism in which eutrophication-driven algal blooms produce hydrophilic organic matter rich in polar groups. This material forms water-soluble complexes with hydrophobic OCPs, enhancing their solubility and promoting upward migration toward the sediment–water interface.

## Figures and Tables

**Table 1:** Changes in OCP concentrations (ng/g) in Lake Qilu sediments from the 1940s to the 1960s.

OCP Class	1940s	1950s–1960s	Fold Change	Trend
∑DDT	1.6	1.9	1.19×	↑
∑Chlordane	0.69	0.61	0.88×	↓
∑Drin	4.8	8.57	1.79×	↑↑
∑Endosulfan	0.9	1.29	1.43×	↑

Note: ∑Drin includes aldrin, dieldrin, and endrin. Arrows indicate the trend of compound class concentrations over time. ↑ indicates an increase; ↓ indicates a decrease; ↑↑ indicates a marked increase (fold change > 1.5×).

## Data Availability

The raw data supporting the conclusions of this article will be made available by the authors upon request.

## Abbreviations

List of abbreviations used in this study.

Abbreviation	Full term
DOM	Dissolved organic matter
FA	Fulvic acid
HIFA	Hydrophilic fulvic acid
HIM	Hydrophilic organic matter
HOFA	Hydrophobic fulvic acid
HOM	Hydrophobic organic matter
OCPs	Organochlorine pesticides
SOM	Sedimentary organic matter
SWI	Sediment–water interface
TN	Total nitrogen
TOC	Total organic carbon
ΣDrin	Sum of aldrin, dieldrin, and endrin
